# Case Report: Refractory mixed acidosis with iatrogenic hyperchloremic acidosis in diabetic ketoacidosis

**DOI:** 10.3389/fendo.2026.1969157

**Published:** 2026-10-21

**Authors:** Jing Lin, Jianjun Zheng, Xiangjin Xu, Chuan Zhou, Guokai Yang, Rongfeng Zhu

**Affiliations:** 1 Department of Endocrinology, The 95th Hospital of Putian, Putian, Fujian, China; 2 Gastroenterology Department, The 95th Hospital of Putian, Putian, Fujian, China; 3 Department of Endocrinology, 900th Hospital of the Joint Logistics Team, Fuzhou, China; 4 Medical Imaging Department, The 95th Hospital of Putian, Putian, Fujian, China; 5 Department of Nephrology, Fujian Provincial Hospital, Shengli Clinical Medical College of Fujian Medical University, Fuzhou, Fujian, China

**Keywords:** continuous renal replacement therapy, diabetic ketoacidosis, hyperchloremic acidosis, mixed acidosis, multiple organ failure

## Abstract

This case highlights a critical but easily overlooked complication of diabetic ketoacidosis (DKA) management—iatrogenic hyperchloremic acidosis superimposed on ketoacidosis—and demonstrates the role of the Δ gap (ΔAG − ΔHCO_3_^-^) in identifying refractory mixed acidosis. The “peripheral metabolic pool” hypothesis is proposed as a possible additional mechanism for persistent pH deterioration despite improvement in central metabolic parameters. A 32-year-old woman with type 2 diabetes presented with severe DKA (pH 6.974) after excessive durian and beer consumption. Despite initial improvement in glucose and ketone levels, her pH paradoxically worsened to 6.84 with progressive hyperchloremia (96.8 to 115 mmol/L), leading to acute heart failure, acute kidney injury*, among others. The Δ gap=ΔAG-ΔHCO_3_^-^<-6* suggested the possible coexistence of high anion-gap ketoacidosis and normal anion-gap hyperchloremic acidosis, consistent with a mixed acidosis. Urinary β_2_-microglobulin >4000 μg/L indicated proximal tubular injury. Continuous renal replacement therapy (CRRT) was initiated at 14 hours after admission; acidosis was successfully corrected after 12 hours of CRRT, with gradual recovery of organ function. Recognition of mixed acidosis and timely individualized CRRT were important in breaking this patient’s vicious cycle. Clinicians should avoid excessive chloride load in DKA management and consider balanced crystalloids after initial resuscitation.

## 1 Introduction

Diabetic ketoacidosis (DKA) is a life-threatening complication of diabetes, characterized by hyperglycemia, ketonemia, and high anion-gap metabolic acidosis. Conventional treatment consists of fluid resuscitation and insulin therapy, aimed at correcting hyperglycemia, ketonemia, and acidosis (1). However, the standard protocol often involves massive infusion of *0.9% sodium chloride*, which has a chloride concentration of 154 mmol/L—far exceeding plasma chloride levels (approximately 98–106 mmol/L). This practice may introduce iatrogenic risks. *0.9% sodium chloride* can induce hyperchloremia, thereby precipitating hyperchloremic metabolic acidosis, particularly when acute kidney injury impairs chloride excretion and further exacerbates acid accumulation. Self et al., in a subgroup analysis of 172 adult DKA patients, found that the median time to DKA resolution was 13.0 hours in the balanced crystalloid group, significantly shorter than 16.9 hours in the *0.9% sodium chloride* group (adjusted HR = 1.68; P = 0.004), suggesting that balanced crystalloids may be the preferred fluid for acute DKA management (2). Li et al., in a frontier debate published in the *New England Journal of Medicine*, also explored the choice between balanced crystalloids and *0.9% sodium chloride* for fluid resuscitation in DKA (3). Furthermore, some patients experience paradoxical worsening of acidosis despite declining blood glucose and ketone levels, suggesting that hyperchloremic acidosis may be superimposed on ketoacidosis, resulting in a mixed acid-base disorder. The delta gap method (ΔAG − ΔHCO_3_^-^) proposed by Wrenn provides a useful approach to identify such mixed acid-base disturbances (4). We herein report a case of severe DKA complicated by iatrogenic hyperchloremic acidosis leading to refractory mixed acidosis and multiorgan failure, and to discuss its possible pathophysiological mechanisms and therapeutic strategies.

## 2 Case description

A 32-year-old Asian female (BMI 25.4 kg/m²) with a 2-year history of type 2 diabetes was admitted to our hospital on April 29, 2024. She had undergone three uncomplicated cesarean sections (2013, 2014, 2019) and had no history of hypertension or coronary heart disease. She presented with a 5-day history of persistent mid-to-upper abdominal pain and a 6-hour history of progressive shortness of breath, nausea, vomiting, and Kussmaul respiration. Three days before admission, she had consumed ~3.6 kg of durian and beer without glucose monitoring. She had been diagnosed with type 2 diabetes for over 2 years with poor medication adherence. Her mother had type 2 diabetes; her father and siblings were healthy. She was married with three children and had regular menstrual cycles. She did not smoke and consumed alcohol occasionally. She had been treated with metformin, repaglinide, linagliptin, and voglibose but did not adhere to the regimen or monitor glucose regularly. Her glycemic control was poor (random glucose ~15 mmol/L), with no prior DKA episodes. Vital signs: pulse 128/min, respiratory rate 36/min, BP 130/88 mmHg, Somnolent state, dehydrated appearance, no rales heard in both lungs, bowel sounds 1–2 times per minute. Finger-stick glucose 32 mmol/L, ketones 3.6 mmol/L, blood pH 6.974, lactate 1.9 mmol/L, base excess –28.5 mmol/L. Serum chloride 96.77 mmol/L, creatinine 56.8 μmol/L. No abnormalities were found in the pancreas on abdominal CT. She was diagnosed with severe DKA.

Fluid resuscitation with 0.9% 0.9% sodium chloride and continuous insulin infusion were started (The maximum insulin infusion rate is 15 units per hour). When the blood glucose level is less than 13.9 mmol/L, infuse 5% glucose solution, and supplement potassium when urine output is present. At 2 hours, glucose dropped to 16.4 mmol/L, ketones 2.4 mmol/L, however, 6 hours later, the patient developed worsening dyspnea and restlessness, with SpO_2_ 88% and a pulse of 134 beats/min. Auscultation revealed diffuse moist rales throughout both lungs. Acute left heart failure was considered, and the symptoms were relieved after furosemide administration. At 9 hours, pH was 6.9, chloride 115 mmol/L, despite cumulative fluid intake 6550 ml and urine output 4600 ml. At 11 hours after admission, 4.2 g of sodium bicarbonate was infused. At 14 hours, she became comatose (pH 6.84, *PaO_2_ 65 mmHg*, bicarbonate 2.6 mmol/L, chloride 115 mmol/L, lactate 1.31 mmol/L). Blood amylase rose to 754.4 U/L, creatinine 207.3 μmol/L, NT-proBNP 8709 pg/mL, *PaO2/FiO2* radio 152.5mmHg, suggesting refractory acidosis complicated by acute heart failure and acute kidney injury. Tracheal intubation and CRRT (CVVHDF mode: Add 11 ml of 10% potassium chloride and 250 ml of 5% sodium bicarbonate to every 4 L of replacement fluid. The ultrafiltration volume is 200–300 ml/h, the replacement volume is 1000–1500 ml/h, the dialysate flow rate is 1000 ml, the blood flow rate is 200 ml/min. Anticoagulation is given with 3000 u of enoxaparin, and an additional 250 u/h is added.) were initiated. After 12 hours of CRRT treatment, the pH returned to normal (7.42). After 25 hours of CRRT treatment, the patient regained consciousness and the tube was successfully removed. Renal function improved (creatinine 69.6 μmol/L by day 15) and she was discharged on day 15. Laboratory data are summarized in **Table 1**, **Figure 1**.

**Table 1:** Selected laboratory data.

Variable	Reference Range, Adults†	1.5 hours before admission (in the Emergency Department)	9 hours after admission	14 hours after admission (before CRRT and tracheal intubation)	26 hours after admission	40 hours after admission (after CRRT disconnection and tracheal tube removal)	2 days after admission
pH Value	7.35-7.45	6.974	6.9	6.84	7.42	7.46	7.47
Bicarbonate	23-29mmol/l		2.9	2.6	21.6	29	26.4
Chloride	98-106mmol/l		115	115	101	96	95
Sodium	136–146 mmol/l		134	132	134	137	133
Potassium	3.5–5.0 mmol/l		4.8	5.0	3.7	3.4	3.8
*Anion Gap*	8-12		16.1	14.4	11.4	12	11.6
*Δ gap=ΔAG-ΔHCO_3_*			-15	-17	-1	7	4
Lactate	0.5-1.6mmol/l	1.9	1.1	1.31	0.87	0.73	1.35
Partial Pressure of Oxygen	83-108mmHg	124	123	65	178	179	179
Partial Pressure of Carbon Dioxide	35-45mmHg		14.7	15.7	33.7	40.8	36.3
Creatinine	41-73umol/l	56.8	34.0	207.3			302.9

†Reference values are affected by many variables, including the patient population and the laboratory methods used. The ranges used at *The 95^th^ Hospital of Putian* are for adults.

who are not pregnant and do not have medical conditions that could affect the results. They may therefore not be appropriate for all patients. *Anion gap = [Na+] - ([Cl-] + [HCO3-]), The calculation of anion gap does not include potassium.*

**Figure 1:**
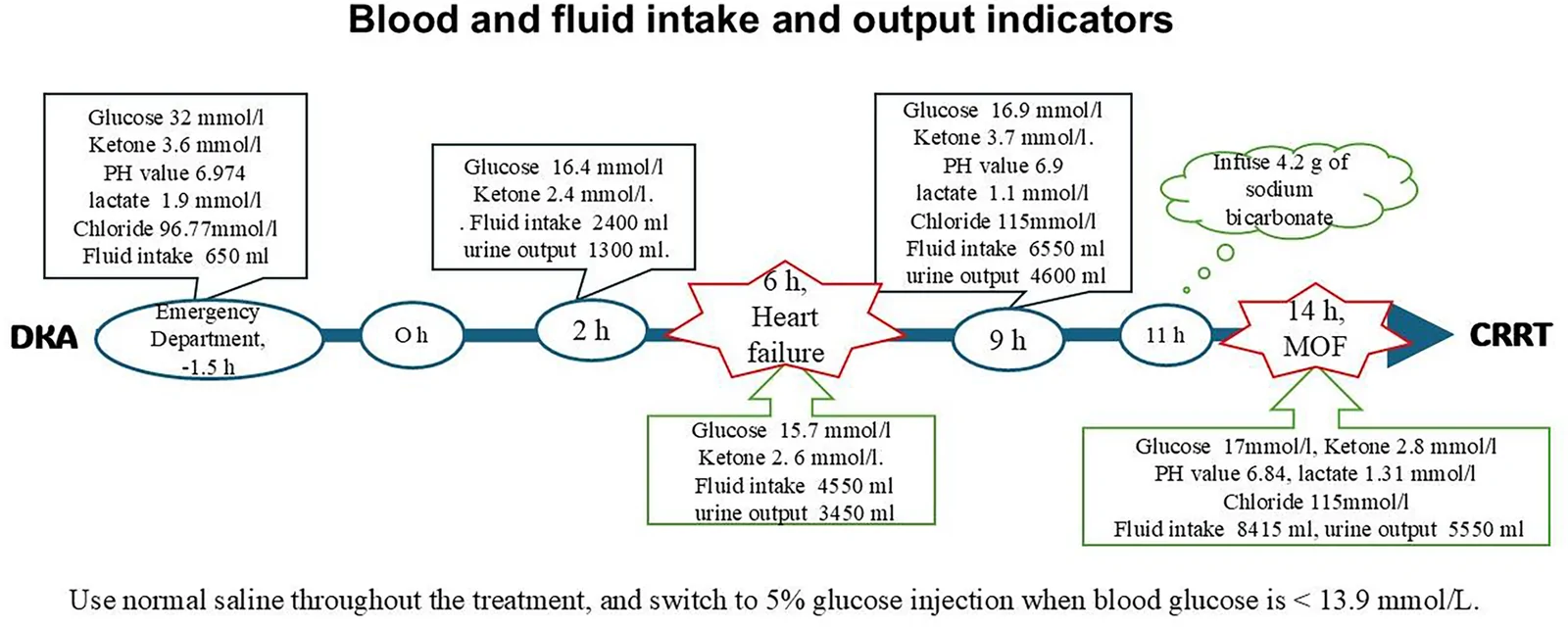
Timeline of fluid balance, glucose, ketones, pH, chloride, lactate, and clinical events.

## 3 Diagnostic assessment

### 3.1 Iatrogenic hyperchloremic acidosis

The patient received exclusively *0.9% sodium chloride* (chloride 154 mmol/L). Serum chloride rose from 96.8 to 115 mmol/L, indicating massive chloride load. Using the Δ gap=ΔAG-ΔHCO_3_^-^: ΔAG = measured AG (16.1) – normal AG (10) =6.1; ΔHCO_3_^-^ = normal HCO_3_^-^ (24) – measured HCO_3_^-^ (2.9) = 21.1; Δ gap =6.1-21.1 = -15. Δ gap<-6 suggests the possible presence of mixed acidosis with coexisting high anion-gap ketoacidosis and normal anion-gap hyperchloremic acidosis (4, 5).

### 3.2 Renal tubular injury

Urinary β_2_-microglobulin >4000 μg/L and serum β_2_-microglobulin 8166.71 μg/L suggest proximal tubular injury, which may impair bicarbonate reabsorption.

### 3.3 Peripheral metabolic pool *(Hypothesis)*

In severe DKA, reduced peripheral tissue perfusion leads to local accumulation of ketones, which are washed back into the circulation after perfusion is restored, causing transient worsening of acidosis. At 14 hours after admission (before CRRT), blood glucose and ketones were lower than on admission; however, after CRRT, blood glucose continued to decrease while ketones paradoxically increased, peaking at 6.2 mmol/L at 9.5 hours after CRRT initiation (**Supplementary Table 3**). This provides indirect support for the hypothesis that improved circulation after CRRT leads to washout of ketones from peripheral tissues back into the circulation.

### 3.4 Exclusion of other causes

The patient had no diarrhea, so gastrointestinal HCO_3_^-^ loss was unlikely; vomiting alone does not cause hyperchloremic acidosis. Although elevated urinary β_2_-microglobulin suggested proximal tubular injury, renal tubular acidosis could not be confirmed because urinary pH, urinary anion gap, and urinary electrolytes were not measured. Acute kidney injury may contribute to hyperchloremic acidosis, but alone it was insufficient to explain the high AG (16.1 mmol/L) or pH 6.84. No history of carbonic anhydrase inhibitors, ammonium chloride, or other chloride-containing drugs was identified. Lactic acidosis was excluded by normal lactate. Methanol/ethylene glycol poisoning was considered unlikely based on the absence of exposure history and lack of compatible clinical features; the osmolal gap was not used because severe lipemia interfered with its calculation. Therefore, the hyperchloremic component was mainly attributed to iatrogenic chloride load, coexisting with ketoacidosis to form mixed acidosis.

## 4 Therapeutic intervention and outcome

CRRT with bicarbonate-buffered replacement fluid improves acid-base and electrolyte disturbances while achieving precise volume control. Acidosis resolved after 12 hours of CRRT, hemodynamics stabilized, and renal function recovered. The patient was discharged on day 15 with normal glucose, lipids, and renal function.

## 5 Discussion

The etiology of DKA is heterogeneous, and clinicians should remain vigilant for atypical triggers and underlying predisposing conditions (6). This case reveals a critical but easily overlooked pathophysiological process in DKA management—iatrogenic hyperchloremic acidosis superimposed on ketoacidosis, leading to refractory mixed acidosis and ultimately multiorgan failure. This recognition has important implications for optimizing clinical management.

### 5.1 Pathophysiology of mixed acidosis

DKA is fundamentally a high anion-gap (AG) metabolic acidosis resulting from overproduction of ketone bodies (mainly β-hydroxybutyrate and acetoacetate) (7). Conventional treatment with insulin and fluid resuscitation typically corrects acidosis. However, massive *0.9% sodium chloride* infusion can fundamentally alter this process. *0.9% sodium chloride* has a chloride concentration of 154 mmol/L, far exceeding plasma chloride (~98–106 mmol/L), and large-volume infusion can cause hyperchloremia. When hyperchloremia is superimposed on pre-existing high-AG acidosis, a “gap” appears between the change in AG (ΔAG) and the fall in bicarbonate (ΔHCO_3_^-^)—i.e., the increase in AG does not fully account for the decrease in HCO_3_^-^. This gap quantitatively represents normal AG hyperchloremic acidosis. Δ gap=ΔAG-ΔHCO_3_^-^<-6 is a reference indicator for mixed acidosis (4, 5). In this case, the difference value was as low as -15, suggesting that two types of acidosis may coexist.

### 5.2 Clinical significance of the peripheral metabolic pool hypothesis

In addition to hyperchloremic acidosis, this case also illustrates another hypothesized mechanism for persistent pH deterioration—the “peripheral metabolic pool” phenomenon. Krausova et al., using sublingual dark-field imaging, demonstrated significant microcirculatory dysfunction in severe DKA, with the degree of impairment strongly correlated with ketone levels (8). This means that when peripheral tissues (muscle, skin) are poorly perfused, locally produced ketones and lactate accumulate. As central glucose and ketone levels decline with treatment, improved peripheral perfusion flushes these sequestered acidic metabolites back into the central circulation, causing transient or persistent acidosis exacerbation. Glaser et al., in a rat model of DKA, further showed that even after systemic pH normalization, neuronal acidosis and neuroinflammation persisted in brain tissue, indicating that tissue-level acidosis recovery lags far behind circulating parameters (9). This hypothesis partially explains the clinical paradox of declining pH despite improving blood glucose and ketone levels (**Figure 2**).

**Figure 2:**
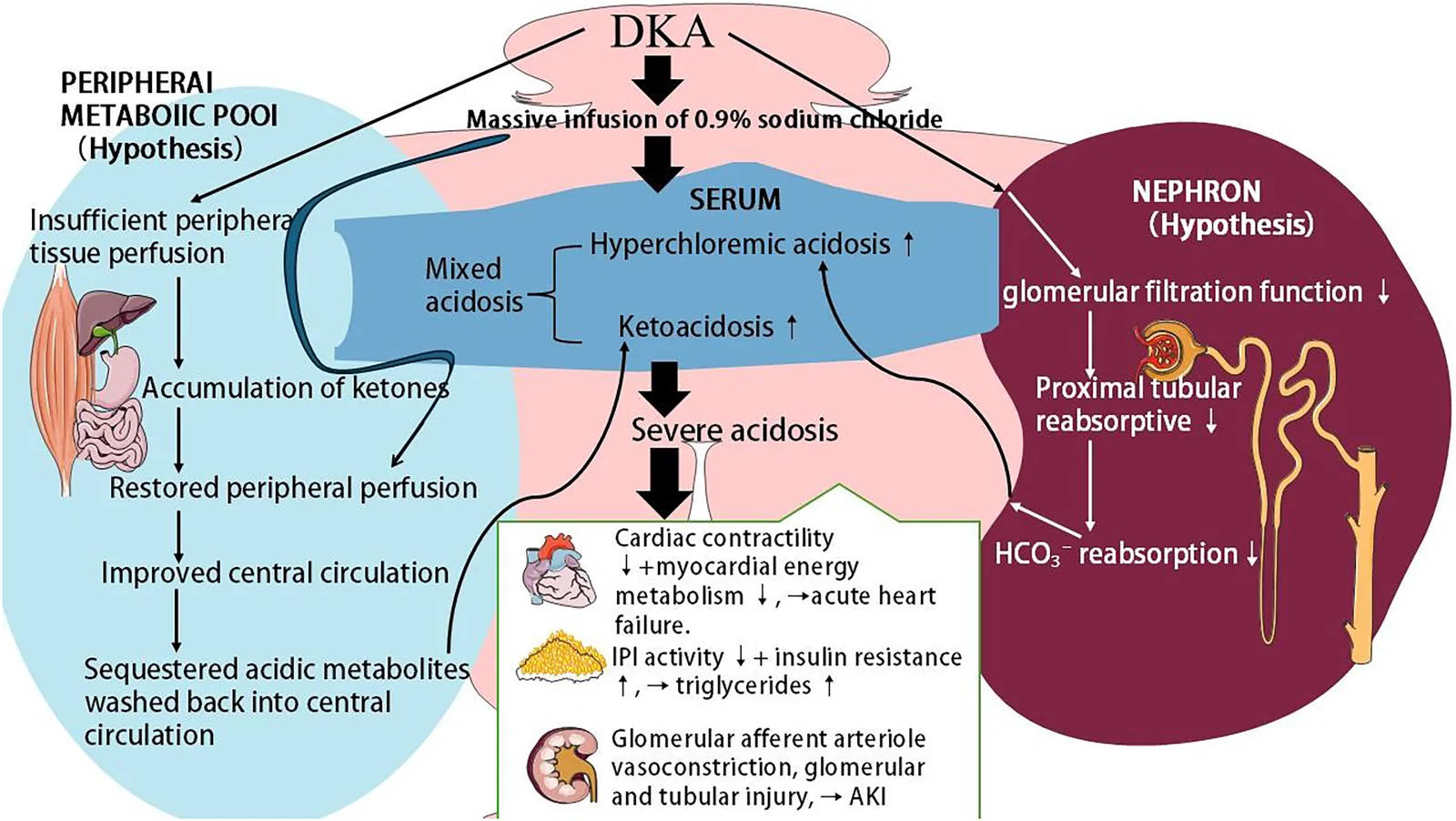
Schematic diagram of mechanisms. This diagram illustrates a *hypothetical mechanism* by which three core pathophysiological processes interact to cause refractory mixed acidosis and subsequent multiorgan dysfunction. Left panel (Peripheral metabolic pool *hypothesis*): In severe DKA states, insufficient peripheral tissue perfusion leads to the accumulation of ketones (particularly β-hydroxybutyrate) and lactate in peripheral tissues, forming a “peripheral metabolic pool.” Following fluid resuscitation and insulin therapy, central circulating glucose and ketone levels decline, while restored peripheral perfusion “washes back” these sequestered acidic metabolites into the central circulation, causing transient or persistent aggravation of acidosis—a mechanism analogous to ischemia-reperfusion injury. Right panel (*Hypothesized mechanisms of iatrogenic hyperchloremic acidosis*): Massive infusion of *0.9% sodium chloride*, combined with acute kidney injury (AKI, serum creatinine from 34 to 302.9 μmol/L), leads to impaired chloride excretion and progressive hyperchloremia (serum Cl^-^ from 96.77 to 115 mmol/L). This results in hyperchloremic normal anion gap acidosis, which superimposes on the original high anion gap ketoacidosis, forming a mixed severe acidosis (*Δ gap=ΔAG-ΔHCO_3_^-^ = -15*). Bottom panel (end-organ effects): The mixed acidosis further triggers: (1) cardiac contractility depression and myocardial energy metabolism impairment, leading to acute heart failure (NT-proBNP 8709 pg/mL); (2) lipoprotein lipase (LPL) activity inhibition and aggravated insulin resistance, exacerbating hypertriglyceridemia; and (3) glomerular afferent arteriole vasoconstriction, along with glomerular and tubular injury, resulting in AKI. Reduced proximal tubular HCO_3_^-^ reabsorption (urine β_2_-microglobulin >4000 μg/L, serum β_2_-MG 8166.71 μg/L) further amplifies the acid load. DKA, diabetic ketoacidosis; LPL, lipoprotein lipase; AKI, acute kidney injury; CRRT, continuous renal replacement therapy.

### 5.3 Hypertriglyceridemia, acute pancreatitis, and DKA: a bidirectional relationship

Severe hypertriglyceridemia (HTG), acute pancreatitis (AP), and DKA can form a vicious cycle. In DKA, insulin deficiency reduces lipoprotein lipase activity and enhances lipolysis, leading to markedly elevated triglycerides. In this patient, TG was 28.21 mmol/L, far exceeding the 11.3 mmol/L threshold for HTG-AP, suggesting that severe HTG was the primary precipitant of AP. Conversely, the inflammatory stress of AP worsens insulin resistance and ketosis, creating a cycle in which DKA aggravates HTG, HTG triggers AP, and AP further exacerbates DKA. Serum lipase and follow-up pancreatic imaging were not obtained in this case; AP diagnosis was based on abdominal pain and amylase 754.4 U/L (approximately 5.6 times the upper limit of normal), meeting two of the Atlanta criteria, though diagnostic certainty is somewhat limited.

### 5.4 Therapeutic value of CRRT in refractory mixed acidosis

When mixed acidosis persists and cardiorenal function is overwhelmed, conventional therapy becomes ineffective. Hyperchloremia not only worsens acidosis but also induces renal afferent arteriolar vasoconstriction, further impairing renal function (10, 11). At this stage, glucose-containing fluids alone cannot effectively reduce serum chloride, and rapid fluid infusion may precipitate heart failure. CRRT with bicarbonate-buffered replacement fluid was associated with correction of hyperchloremic acidosis and precise volume control. Following CRRT initiation, acidosis was corrected and circulation stabilized in this patient; however, causality cannot be inferred from a single case. These observations suggest a temporal association, but this single case cannot establish a specific causal mechanism.

## 6 Clinical implications

This case highlights the need for vigilance against mixed acidosis in DKA management and provides the following practical recommendations: First, when blood glucose and ketone levels decline but pH does not improve or even worsens, the value of Δ gap = ΔAG - ΔHCO_3_ can be calculated. If the difference is less than -6 to 8, it indicates mixed acidosis, warranting adjustment of the fluid strategy and CRRT should be considered in light of the patient’s unique circumstances. However, it should not be used as a sole indication for CRRT. Second, after initial resuscitation, balanced crystalloids with lower chloride content (e.g., Plasma-Lyte or lactated Ringer’s) should be preferred to avoid iatrogenic acidosis (12–14). Finally, for patients with delayed urine output after CRRT, urinary β_2_-MG and other tubular injury markers should be measured to assess renal tubular reserve. The successful outcome in this case demonstrates that *recognition* and aggressive intervention for mixed acidosis are key to improving prognosis in DKA patients with multiorgan failure.

## 7 Patient perspective

The patient had no recollection of the acute event and expressed gratitude for the care. She has since adhered to a diabetic diet and regular glucose monitoring.

## 8 Diagnostic challenges, prognosis, and limitations

Diagnostic challenges include the need for high-quality evidence to support the identification of mixed acidosis when the Δ gap=ΔAG-ΔHCO_3_^-^<-6, the failure to measure 24-hour urinary electrolytes and urinary titratable acid, and the lack of serum lipase testing and subsequent pancreatic imaging. The short-term prognosis was favorable, with recovery of organ function at discharge; however, long-term follow-up showed renal and metabolic outcomes returned to normal. Intervention adjustments included early initiation of CRRT and other measures. Pre-admission treatment adherence was poor, but tolerance during hospitalization was good. Adverse events included acute heart failure, AKI, and acute pancreatitis, which may have been aggravated by fluid overload; failure to switch to balanced crystalloid in a timely manner was also a therapeutic deficiency. Limitations include the single-case design and retrospective data. These observations suggest a temporal association, but this single case cannot establish a specific causal mechanism.

## Data Availability

The original contributions presented in the study are included in the article/**Supplementary Material**. Further inquiries can be directed to the corresponding author.
